# Predicting early-stage coronary artery disease using machine learning and routine clinical biomarkers improved by augmented virtual data

**DOI:** 10.1093/ehjdh/ztae049

**Published:** 2024-08-09

**Authors:** Angela Koloi, Vasileios S Loukas, Cillian Hourican, Antonis I Sakellarios, Rick Quax, Pashupati P Mishra, Terho Lehtimäki, Olli T Raitakari, Costas Papaloukas, Jos A Bosch, Winfried März, Dimitrios I Fotiadis

**Affiliations:** Unit of Medical Technology and Intelligent Information Systems, Department of Materials Science and Engineering, University of Ioannina, Ioannina, Greece; Department of Biological Applications and Technology, University of Ioannina, Ioannina, Greece; Department of Clinical Psychology, University of Amsterdam, Amsterdam, The Netherlands; Unit of Medical Technology and Intelligent Information Systems, Department of Materials Science and Engineering, University of Ioannina, Ioannina, Greece; Computational Science Lab, Institute of Informatics, University of Amsterdam, Amsterdam, The Netherlands; Unit of Medical Technology and Intelligent Information Systems, Department of Materials Science and Engineering, University of Ioannina, Ioannina, Greece; Biomedical Engineering of the Department of Mechanical Engineering and Aeronautics, University of Patras, Patras, Greece; Computational Science Lab, Institute of Informatics, University of Amsterdam, Amsterdam, The Netherlands; Department of Clinical Chemistry, Faculty of Medicine and Health Technology, Tampere University, Tampere, Finland; Faculty of Medicine and Health Technology, Finnish Cardiovascular Research Center Tampere, Tampere University, Tampere, Finland; Department of Clinical Chemistry, Fimlab Laboratories, Tampere, Finland; Department of Clinical Chemistry, Faculty of Medicine and Health Technology, Tampere University, Tampere, Finland; Faculty of Medicine and Health Technology, Finnish Cardiovascular Research Center Tampere, Tampere University, Tampere, Finland; Department of Clinical Chemistry, Fimlab Laboratories, Tampere, Finland; Research Centre of Applied and Preventive Cardiovascular Medicine, University of Turku, Turku, Finland; Department of Clinical Physiology and Nuclear Medicine, Turku University Hospital, Turku, Finland; Centre for Population Health Research, University of Turku and Turku University Hospital, Turku, Finland; Department of Biological Applications and Technology, University of Ioannina, Ioannina, Greece; Department of Clinical Psychology, University of Amsterdam, Amsterdam, The Netherlands; Department of Internal Medicine V, University of Heidelberg, Mannheim, Germany; Clinical Institute of Medical and Chemical Laboratory Diagnostics, Medical University of Graz, Austria; SYNLAB Holding Deutschland GmbH, Augsburg, Germany; Unit of Medical Technology and Intelligent Information Systems, Department of Materials Science and Engineering, University of Ioannina, Ioannina, Greece; Department of Biomedical Research, FORTH-IMBB, GR 45110 Ioannina, Greece

**Keywords:** Coronary artery disease, Machine learning, Classification algorithms, Data Augmentation

## Abstract

**Aims:**

Coronary artery disease (CAD) is a highly prevalent disease with modifiable risk factors. In patients with suspected obstructive CAD, evaluating the pre-test probability model is crucial for diagnosis, although its accuracy remains controversial. Machine learning (ML) predictive models can help clinicians detect CAD early and improve outcomes. This study aimed to identify early-stage CAD using ML in conjunction with a panel of clinical and laboratory tests.

**Methods and results:**

The study sample included 3316 patients enrolled in the Ludwigshafen Risk and Cardiovascular Health (LURIC) study. A comprehensive array of attributes was considered, and an ML pipeline was developed. Subsequently, we utilized five approaches to generating high-quality virtual patient data to improve the performance of the artificial intelligence models. An extension study was carried out using data from the Young Finns Study (YFS) to assess the results’ generalizability. Upon applying virtual augmented data, accuracy increased by approximately 5%, from 0.75 to –0.79 for random forests (RFs), and from 0.76 to –0.80 for Gradient Boosting (GB). Sensitivity showed a significant boost for RFs, rising by about 9.4% (0.81–0.89), while GB exhibited a 4.8% increase (0.83–0.87). Specificity showed a significant boost for RFs, rising by ∼24% (from 0.55 to 0.70), while GB exhibited a 37% increase (from 0.51 to 0.74). The extension analysis aligned with the initial study.

**Conclusion:**

Accurate predictions of angiographic CAD can be obtained using a set of routine laboratory markers, age, sex, and smoking status, holding the potential to limit the need for invasive diagnostic techniques. The extension analysis in the YFS demonstrated the potential of these findings in a younger population, and it confirmed applicability to atherosclerotic vascular disease.

## Introduction

Atherosclerosis, characterized by an inflammatory condition that causes deposits of lipids and tissue scarring in the vessel wall and artery narrowing (stenosis), stands as a primary aetiology of coronary artery disease (CAD), myocardial infarction, and heightened mortality rates.^1^ CAD is the most common type of heart disease, affecting millions worldwide. About 20.5 million US adults have CAD, according to the Centres for Disease Control and Prevention.^2^

A number of modifiable lifestyle and intervention-related risk factors are linked to CAD; therefore, timely detection and accurate diagnosis are essential for successful clinical management. Recognizing the importance of early diagnosis in improving patient outcomes, early detection can prevent disease progression and streamline treatment. Invasive coronary angiography (ICA) is a gold standard procedure for assessing CAD.^3^ Before stenoses are detected with ICA, the disease may already be advanced. This shortens the time window for early intervention in asymptomatic individuals, while angiographies may produce uncertain results or are conducted in people with mild illness, highlighting the need for further development and validation of reliable alternatives for early CAD screening.

In light of these challenges, multiple biomarker monitoring emerges as a promising avenue for enhancing cardiovascular event prediction.^4,5^ Examples of compound risk assessment scores are the Framingham Risk Score^6,7^ and the American College of Cardiology/American Heart Association 2013 risk score (ACC/AHA13).^8^ These risk scores involve a combination of clinical variables and blood chemistry measures. The efficacy of the compound indexes is enhanced by the fact that none of the risk variables included in the aggregate scores are separately sufficiently accurate in predicting incident or prevalent CAD when assessed. However, despite their utility, there remains a notable gap: the absence of a non-invasive tool to assess pre-test probability of obstructive CAD effectively. The current guidelines recommend estimation of the pre-test probability of CAD scores to guide decisions on whether diagnostic testing could be deferred or performed, and whether the initial test should be non-invasive or invasive.^9^ However, recent studies have shown that the performance of the traditional pre-test probability of CAD models is limited in estimation of obstructive CAD.^10^ Because angiography may pose risks, it is typically reserved for cases where CAD is suspected, potentially missing early-stage patients and those who are asymptomatic. Herein lies the potential of machine learning (ML), an advance analytical tool with the capacity to produce unbiased risk assessments based on empirical data, which provides a potentially feasible approach to arriving at such a score.^11^ An ML test, based on a combination of blood tests and clinical data, could offer a non-invasive means of identifying patients at earlier stages.

Therefore, the objective of the present study is to develop an ML-based model that can diagnose early CAD utilizing a limited set of laboratory results, sex, age, and smoking status, i.e. a compound score that can be determined using a single blood assessment. We intended to capitalize on laboratory data that may be routinely collected in order to enable the affordable application of a diagnostic score in clinical practice. The present study additionally introduces ‘virtual population creation’ as a methodological innovation to optimize the ML-based risk score. A computational technique called virtual population creation is used to create synthetic patient data that closely resemble real-world distributions. Its purpose is to enhance the statistical power of clinical research databases with limited sample sizes. The performance of artificial intelligence (AI) models can be improved by combining real and virtual patient data.^12^ We investigated if using virtual population data can improve the performance of CAD classification algorithms.

## Methods

### Dataset

The Ludwigshafen Risk and Cardiovascular Health (LURIC) cohort study was used in this study. LURIC is a cohort of patients who underwent coronary angiography between 1997 and 2001 in order to identify environmental, biochemical, and genetic risk factors for cardiovascular disease (CVD). A total of 3316 patient records were included. Inclusion criteria were as follows: German ancestry (to limit genetic heterogeneity), clinical stability, and availability of a coronary angiogram. Patients with acute coronary syndromes (ASCs) were included only if they were clinically stable at the time of the angiogram. Clinically unstable patients, such as those with ongoing chest pain, haemodynamic instability, or other signs of active ischaemia, were excluded. Exclusion criteria were acute illness besides ASCs, chronic diseases other than non-cardiac diseases, and a history of cancer. The exclusion criteria were determined a priori to minimize the impact of co-existing non-CVD on intermediate biochemical phenotypes or clinical prognosis (limitation of heterogeneity). More information can be found in the Supplementary material online, Table S1. Prior studies have included demographic information and other background information about the patients.^13,14^

We performed an extension analysis using data from the Young Finns Study (YFS), a longitudinal study of cardiovascular risk factors conducted in Finland from childhood to adulthood. Commencing in 1980 with a sample of 3596 children and adolescents aged 3–18 years, the YFS has continued for over 40 years to explore the impact of childhood factors on the risk of cardiovascular diseases in adulthood.^15^ The 2001 follow-up, which included 2283 participants aged 24–39 years, was used for the extension analysis.

### Methodology

**Figure 1:**
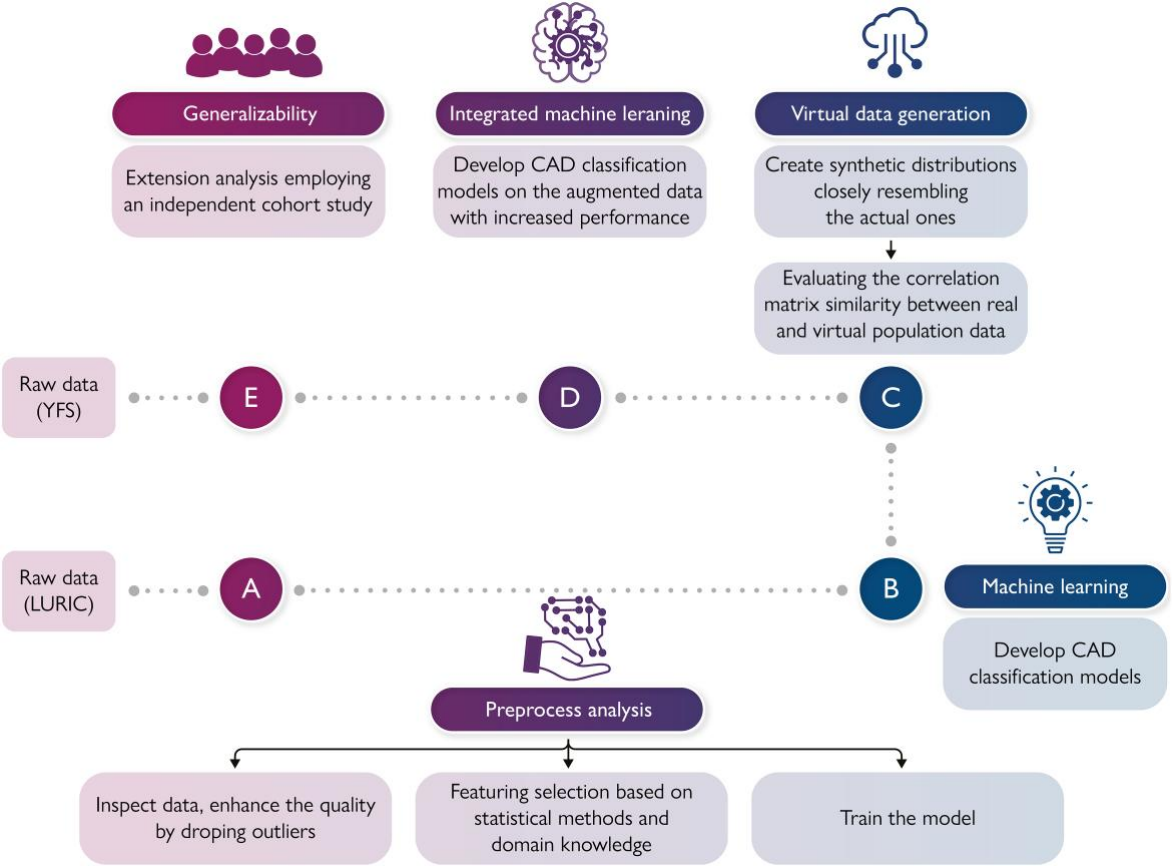
Development of a machine learning–based algorithm to screen for angiographic coronary artery disease and cerebrovascular disease. Data from the Ludwigshafen Risk and Cardiovascular Health Study were used. They underwent a workflow of data curation, feature engineering, model selection and training, feature importance analysis, and explainable analysis (A and B). Virtual data generation was performed to enhance disease classification models (C and D). Additionally, an extension analysis using an ongoing multicentre follow-up study, called Young Finns study, was conducted to ensure reliable results (E). A machine learning pipeline was created that allows screening for angiographic coronary artery calcification (CAC) and cardiovascular disease with high sensitivity and specificity.

Our analyses involved the following three steps: first, to employ advanced ML algorithms to accurately identify and remove outliers, while simultaneously improving the quality of the input data through attribute selection and feature engineering; secondly, to augment the size of the database by integrating virtual data with the pre-existing clinical data; and finally, to develop an integrated supervised ML model that utilizes both real and virtual data to achieve an optimal classification of patients with CAD. An extension analysis using the YFS was conducted to demonstrate the relevance of these findings in a younger demographic, confirming their applicability to atherosclerotic vascular disease. The overall pipeline of methodology is illustrated in *Figure 1* (Graphical Abstract). The initial set of attributes encompassed demographic information, molecular markers, inflammatory markers, and lipid omics markers (for the full list, see Supplementary materials, Initial attributes). The workflow involved curation of clinical data and feature engineering (*Figure 1A*). Following feature selection and incorporating domain expertise, laboratory data, as well as age, sex, and smoking status, were utilized. Subsequently, the selection of the ML algorithm model training, model evaluation, feature importance analysis, and explainable analysis were made (*Figure 1B*). The next step (*Figure 1C*) was the virtual data generation with high similarity with the real ones and the use of the augmented data as input to an Integrated ML (*Figure 1D*) to assess whether the aggregation of real with virtual patient data can improve the performance of the existing disease classification models. Furthermore, to ensure the consistency of the obtained results, an extension analysis (*Figure 1E*) was conducted using an ongoing multicentre follow-up study, the YFS. The aim of this investigation was to assess and verify the performance of the developed models.

#### Data pre-processing

For missing values, we imputed features using the k-nearest neighbour method for the continuous and the most common value for categorical features.^16^ For reference, see *Table 1* to ascertain the extent of missingness in the data. We performed anomaly detection to identify outliers using ML techniques such as Isolation Forest and Local Outlier Factor.^17^ These methods detect outliers based on how much the data points deviate from most of the dataset and are particularly useful for high-dimensional datasets. To minimize dimensionality and prevent overfitting, we employed a hybrid strategy combining feature ranking methods with domain expertise. We first used the Pearson correlation coefficient to measure linear correlation,^18^ and then used feature ranking algorithms such as Information Gain, Mutual Information, and χ^2^ tests to rank input features.^19,20^ We also applied a multivariate feature selection method to account for class relevance and dependency between feature pairs.^21^ Based on the features’ importance in several algorithms and our experts’ clinical expertise, we selected the most important ones. This approach allowed us to develop an accurate predictive model for prevalent CAD as defined by at least one stenosis equal or greater than 20% on coronary angiography. This threshold, selected to prioritize early-stage disease detection, guided the identification of key features while systematically eliminating irrelevant or redundant variables.

**Table 1:** Selected attributes subsequent to attribute selection from the LURIC and YFS databases, accompanied by their respective descriptive statistics. Notable attributes include carotid intima-media thickness (IMT) and coronary artery disease (CAD)

Attribute type	Attributes	LURIC		YFS	
		Mean (±standard deviation)	Missingness (%)	Mean (±standard deviation)	Missingness (%)
**Demographic**	Age	63 (±10.62)	0	31 (±5)	0
	Gender	Male 2310, Female 1006	0	Female 1832, Male 1764	0
**Clinical/laboratory**	Apolipoprotein A-I	129.4 (±24.9) (mg/dL)	0.030157	150 (±20) (mg/dL)	36.5
	Troponin T	0.08 (±0.41) (ng/mL)	3.256936	0.00403 ng/mL (±0.0018)	36.5
	Smoking status	No: 1194, Ex: 1468, Active: 654	0	less often than daily or never: 1906, Daily: 641,	29.1
	Lipoprotein(a)	29.22 (±34.73) (mg/dL)	0.090470	12.4(±12.1) mg/dL	
	ACE	25.41(±15.85) (U/L)	1.447527	0.04(±0.02) mmol/L	37.7
	Haptoglobin	158.89 (±75.31) (mg/dL)	0.482509	Not available	Not available
	CAD/carotid IMT	Yes: 2583, No: 733	0	Yes: 295, No: 1970	37

Selected attributes subsequent to attribute selection from the LURIC and YFS databases, accompanied by their respective descriptive statistics. Notable attributes include carotid intima-media thickness (IMT) and coronary artery disease (CAD).

#### Supervised machine learning

The problem of predicting CAD has been framed as a binary classification task based on the presence or absence of CAD. Out of the 3316 patients who underwent coronary angiography, 2583 were diagnosed with CAD, while the remaining 733 were identified as healthy. Based on the pre-processed dataset's features, we selected two distinct ML classifiers, namely, random forests (RFs)^22^ and gradient boosting (GB),^23^ for our analysis. Considering the imbalanced number of cases in each class we decided to partition the dataset into a training and a testing set in a manner that preserves the original proportions of cases in the two classes. Such a partitioning is commonly referred to as a stratified train–test split. Class imbalance handling was used to address the population imbalance between the control and target groups using random down sampling with replacement on the control group. Ten random executions of the method were performed, and the average of the results was obtained. It is well known that as the number of possible predictive attributes rises, overfitting of the models due to their complexity can produce implausible results. In order to attain the best results, a grid search for parameter tuning was utilized for the prediction effect. This was accomplished by optimizing the model for the particular data to adapt to varied degrees. An externally stratified 10-fold cross-validation was used to gauge the proposed method's classification performance. Stratification ensures that the class distribution is maintained in the dataset for each validation step. Evaluation metrics were used to assess the performance of the model; for further details, see Supplementary material online, Supplementary methods, Evaluation metrics. Explainable AI (XAI) techniques were integrated to enhance model interpretability.^24^ Shapley values—a crucial part of XAI—were calculated to determine the extent to which each attribute contributed to the model's predictions.^25^ These values provided insights into feature importance and aided in unravelling the complex relationships between features.

#### Virtual data generation

Virtual population creation is a computational method used to produce synthetic patient data that mimics real-world distributions.^23^ It improves the statistical power of clinical research databases with limited sample sizes. We employed five advanced techniques for creating high-quality virtual patient data. We ensured equal numbers of control and target groups to avoid class imbalance issues (total size 5166). To enhance resemblance to real patients, we employed advanced virtual data generation methods, including Tabular Preset, Copulas (encompassing Gaussian Copula and Copula Generative Adversarial Network), and Deep Learning–driven models like Conditional Tabular Generative Adversarial Network and Tabular Variational Autoencoder -based deep learning model.^26^ For details on methods to generate virtual data, refer to Supplementary material online, Supplementary methods, to generate virtual data. For each technique, we compared the distribution of virtual attributes with the real attributes, which allowed us to assess the similarity of distribution patterns. To measure the similarity, we utilized statistical tests, including the Kolmogorov–Smirnov (K–S) test,^27^ which compares the distributions of two datasets. In our case, we used this test to compare the distribution of the virtual attributes with their real counterparts. Additionally, we calculated correlation and absolute differences between the synthetic and real data. Spearman rank-order correlation coefficient,^28^ a measure of monotonicity in the relationship between two datasets, was utilized to evaluate the correlation.

#### Supervised integrated machine learning

The GB and RFs algorithms were employed in conjunction to evaluate the influence of data augmentation on classification performance. Real and synthetic patient data were combined into one single dataset on which both algorithms were trained. Following the training of the algorithms on the augmented dataset, the algorithms were tested using a 10-fold cross-validation technique to ensure the robustness and reliability of the results.

#### Extension

To assess the generalizability of our findings, we performed an extension analysis using the YFS. The YFS started in 1980 with 3596 children and adolescents aged 3–18 years and has continued for over 40 years to assess the impact of childhood factors on the risk of cardiovascular diseases in adulthood.^15^ For the extension analysis, we used a rigorous approach, utilizing just the most significant features discovered in the prior study based on feedback from domain experts and feature selection algorithms. This ensured that the most relevant and informative features were used as input data. We followed a pipeline similar to the previous one. The attributes aligned with those identified in the LURIC study, except for haptoglobin, which was not available in the YFS cohort. After that, we used ML classifiers, and we assessed the outcomes using standard performance criteria. In this context, the target variable encompassed participants displaying Bulbus plaque and/or carotid intima-media thickness (IMT) measurements surpassing the 90th percentile, or bulbus IMT surpassing the 90th percentile. Notably, this focus leaned towards atherosclerotic vascular disease. This particular emphasis underlines the nature of this analysis as an extension specifically designed to explore the broader spectrum of cardiovascular health beyond CAD, as investigated in the original study.

## Results

We started with a broad range of attributes, including clinical, molecular, and laboratory markers, demographic data, lipidomics, and behavioural factors. After incorporating domain knowledge information and applying feature selection and statistical techniques, we arrived at a more restricted set of eight attributes for our analysis: age, gender, smoking status, haptoglobin, lipoprotein(a), troponin T, apolipoprotein A-I, and angiotensin-converting enzyme (ACE) (*Table 1*).

### Base machine learning model

**Figure 2:**
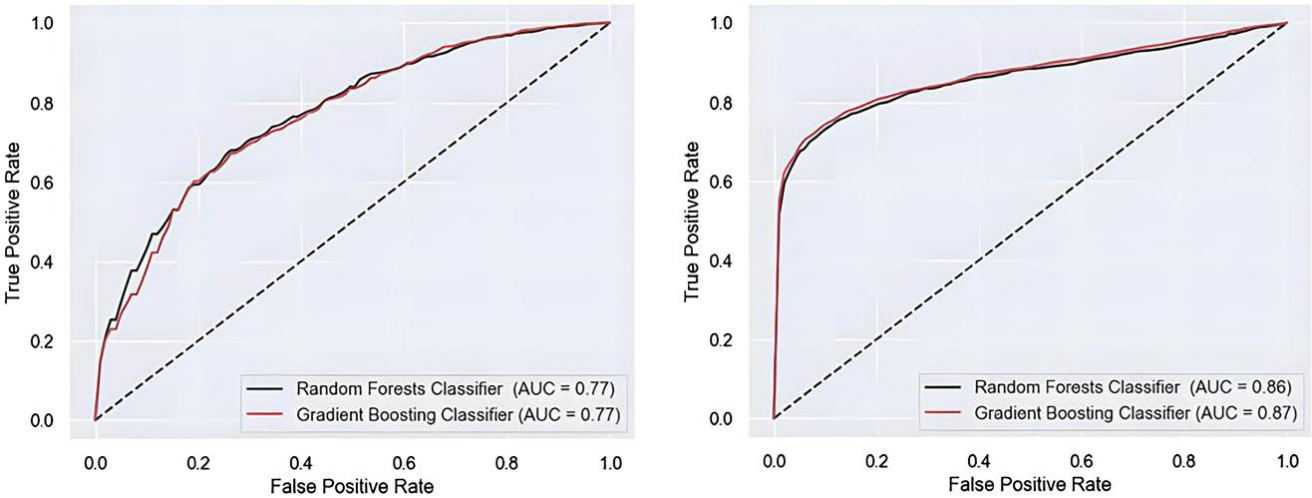
Receiver operating curve along with their respective area under the curve values for gradient boosting and random forests of the base model (left). Receiver operating curve curve depicting area under the curve values for the integrated coronary artery disease prediction models (right). The area under the curve for each classifier is given nearer to the classifier model name.

The model performance evaluation includes several metrics, namely, accuracy, sensitivity, specificity, area under the receiver operating curve (ROC), and precision. The average accuracy of the RFs classifier was found to be 0.75, with a sensitivity of 0.81, a specificity of 0.55, an area under the curve (AUC) value of 0.77, and a precision of 0.86. Application of the GB algorithm to the data resulted in the following performance metrics: accuracy of 0.76, sensitivity of 0.83, specificity of 0.51, AUC of 0.77, and precision 0.85, (*Table 2*, Base model). To further improve the results, we developed an integrated model and a data generation approach to capitalize on the possible synergies between the classifiers and the benefits of enhanced data representation. The application of the GB algorithm to the data resulted in the following performance metrics: *Figure 2* (left) displays the ROC for both classifiers, along with their respective AUC values.

**Table 2:** Performance metrics (accuracy, sensitivity, specificity, AUC ROC, and precision) of different models at various steps: the base model (RFs and GB), the integrated model (with augmented data), and the extension model (on an independent cohort)

Step	Base model		Integrated model (augmented data)		Extension model (independent cohort)	
	RFs	GB	RFs	GB	RFs	GB
Accuracy	0.75	0.76	0.79	0.80	0.72	0.73
Sensitivity	0.81	0.83	0.89	0.87	0.88	0.79
Specificity	0.55	0.51	0.70	0.74	0.61	0.69
AUC ROC	0.77	0.77	0.86	0.87	0.83	0.82
Precision	0.86	0.85	0.79	0.82	0.61	0.64

Performance metrics (accuracy, sensitivity, specificity, AUC ROC, and precision) of different models at various steps: the base model (RFs and GB), the integrated model (with augmented data), and the extension model (on an independent cohort).

RFs, random forests; GB, gradient boosting.

Additionally, a SHapley Additive exPlanations (SHAP) analysis was carried out to compute the contribution of each feature to the outcome. The findings underline the significance of age and gender as risk factors for CAD, as well as the importance of laboratory analyses. A high lipoprotein(a) concentration, for instance, is positively associated with angiographic CAD. Haptoglobin is negatively correlated with CAD (*Figure 4*).

### Augmented data integrated machine learning model

We employed five approaches to generate virtual patient data based on the attributes selected from the LURIC study. The K–S test results, with scores between 0.8 and 0.98, indicated a high degree of similarity between the distributions, affirming the effectiveness of the Gaussian Copula generator. The Spearman rank-order correlation coefficient was 0.96, with a corresponding *P*-value of 1.008*e*–19. To provide a comprehensive evaluation, we used various visualizations, including density plots, kernel density, and violin plots, comparing the original and synthetic data. These results and visualizations can be found in the Supplementary material online, Figures S1–S4.

**Figure 3:**
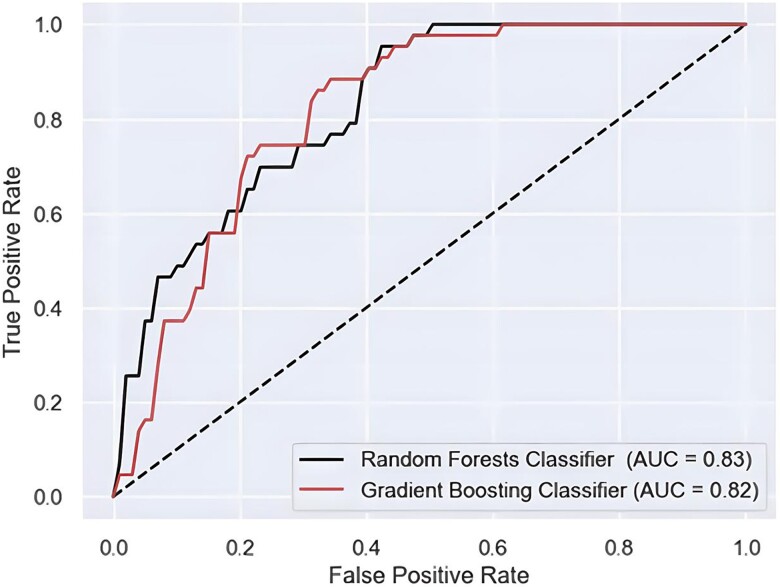
The receiver operating curve curve illustrates the area under the curve values for gradient boosting and random forests in the extended model, further highlighting its predictive performance.

The aggregation of both real and virtual data showcased a significant increase in specificity for CAD (*Table 2*, Integrated model). More specifically, the application of the GB algorithm to the dataset yielded the following performance metrics: accuracy 0.80, sensitivity 0.87, specificity 0.74, an AUC 0.87, and precision 0.82. The RFs classifier exhibited an average accuracy of 0.79, a sensitivity of 0.89, a specificity of 0.70, an AUC value of 0.86, and a precision of 0.79, both significant enhancement in specificity. *Figure 3* displays the ROC for both classifiers, along with their respective AUC values. Performance metrics for each fold, mean values, and 95% confidence intervals can be found in the Supplementary material online, Tables S2 and S3.

### Extension

**Figure 4:**
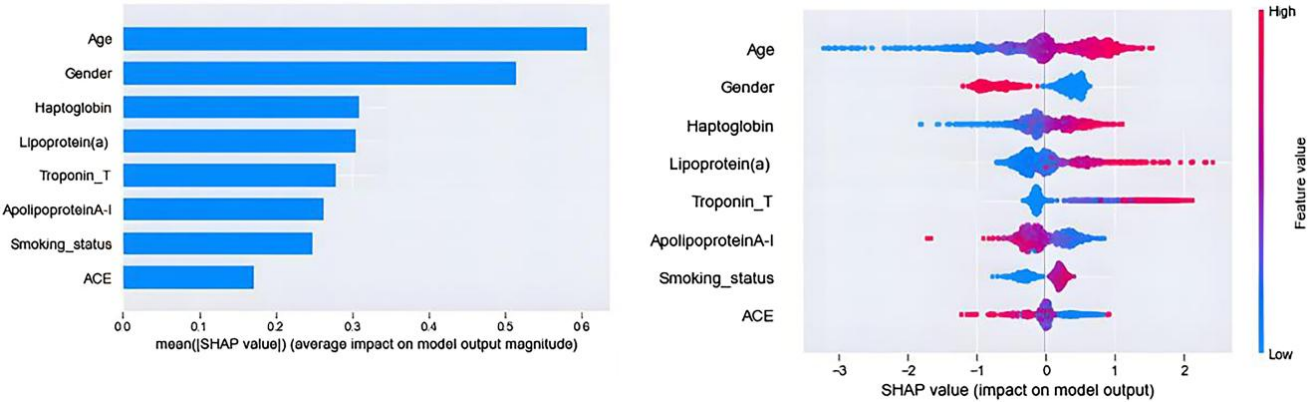
Importance of individual features for the distinction of coronary artery disease from controls. Left panel: feature importance using bar plots of mean SHapley Additive exPlanations (SHAP) values. Right panel: contributions of individual input attributes, either positive or negative. The plot includes all data points from the training set, providing the following: (i) Feature importance, which ranks variables from least to most significant; (ii) Impact, indicated by the horizontal position of each point, showing whether the corresponding characteristic has a positive or negative effect on the prediction; (iii) Original value is shown by the colour of each point; for a certain feature, blue denotes low values and red, high values.

The selected attributes along with their corresponding statistical descriptions of the extension analysis are listed in *Table 1*. The application of the GB on the data yielded: accuracy = 0.73; sensitivity = 0.79; specificity = 0.69; AUC = 0.82; and precision = 0.64. The average accuracy of the RFs classifier was found to be 0.72, while its sensitivity and specificity were 0.88 and 0.61, with a precision of 0.61 (*Table 2*, Extension model). The AUC values for both classifiers are given in *Figure 4*. The ROC curve indicates that RFs classifier achieves a 0.83 AUC in detecting atherosclerotic disease of the carotids.

## Discussion

In our study aimed at detecting early-stage CAD using ML, we used data from LURIC study. Through the integration of virtual patient data our ML models exhibited improvements in accuracy, sensitivity, and specificity. Moreover, validation in a younger population from the YFS reaffirmed the robustness and generalizability of our findings, emphasizing the potential applicability of ML-based approaches across diverse patient demographics for CAD diagnosis and risk assessment.

Many approaches have been developed to predict CAD and pinpoint important risk variables that are associated with it. Most of these studies focus on future CAD-related outcomes and use conventional statistical analysis or ML classification algorithms with non-imaging and imaging input data. Prior ML-based studies have explored CAD prediction and its progression, besides discovering significant predictors.^29^ An examination of the relationship between routine blood test results and the risk of future development of CAD, followed by the construction of a two-layer Gradient Boosting Decision Tree model based on ordinary blood samples to predict the risk of CAD.^30^ The main goal of Kigka *et al.*^31^ was to develop an ML predictive model that combines baseline characteristics of typical patients with non-invasive imaging data from Computed Tomography Coronary Angiography to predict the risk obstructive CAD after six years. Velusamy *et al.* developed an ensemble of multiple classifiers, including K-Nearest Neighbour, Random Forest Ensemble, and support vector machine (SVM), to detect and forecast CAD at an early stage. The ensemble model combines class probabilities using average-voting, majority-voting, and weighted-average voting (WAVEn) for accurate CAD prediction. The statistical analysis revealed the reliability of the WAVEn algorithm in accurately distinguishing between individuals with CAD and those who are healthy.^32^ A comparison of the positive predictive value of CAD using artificial neural network (ANN) and SVM algorithms was implemented by Ayatollahi *et al.*^33^ demonstrating that SVM has higher accuracy and sensitivity, implying its superior performance in CAD prediction compared with ANN, emphasizing the potential for further exploration of diverse data mining algorithms to improve disease prediction.

The present study had two main objectives: (i) utilize advanced ML techniques and routine laboratory tests for early-stage CAD detection, and (ii) investigate the potential of improvement in classification accuracy for CAD identification by combining real patient data with generated virtual data. They were achieved through a stepwise workflow that including data curation, domain knowledge integration, and the use of ML algorithms (GB and RFs). Random forests and GB were chosen for this study because they have proven to be useful in classification problems, notably in healthcare.^34,35^ Random forests excel in handling complex interactions between variables and providing insights into feature importance, which are critical for our heterogeneous dataset. GB, on the other hand, increases predictive performance by learning from errors, making it ideal for increasing CAD identification accuracy.^23^

Our key findings reveal the superior performance of the GB algorithm over the RFs, showcasing improved accuracy and sensitivity, consistent with existing ML literature.^36,37^. Augmenting data using Gaussian Copula significantly improved specificity in CAD classification for both GB and RFs. Moreover, our approach's potential was strengthened by an extension analysis, which proved its applicability to atherosclerotic vascular disease in a younger population. We mainly wished to use a limited array of commonly and conveniently available laboratory tests. We also generated a virtual population with high similarity and correlation to real patients and showed that the use of augmented data significantly improved the performance of our models. The superiority of GB is attributed to the algorithm's sequential training process, which progressively corrects errors, and its proficiency in utilizing weak learners to effectively capture intricate data patterns.^23^ Furthermore, the integration of augmented data resulted in an improved specificity for CAD classification on both GB and RFs. The YFS extension affirmed the findings’ applicability to a younger population, despite a different readout from angiographic CAD, verifying that our model is applicable to atherosclerotic vascular disease. These findings highlight the effectiveness of our suggested methodology in predicting prevalent CAD and demonstrate the potential of integrating virtual data to enhance model performance. We also examined the contributions of each feature to the diagnosis of early CAD in the proposed ML model. Our results emphasize the significance of age, gender, and smoking status as risk factors for CAD and imply that laboratory tests such as lipoprotein(a), troponin T, ACE, and haptoglobin may have potential for identifying patients with CAD. While age, gender, and smoking status have been traditionally recognized as crucial risk factors for CAD, the role of laboratory tests such as lipoprotein(a), troponin T, ACE, and haptoglobin in CAD prediction has garnered increasing attention in recent research.^38–40^ Numerous studies have investigated the association between ACE levels and CAD risk.^41,42^ ACE plays a central role in the renin–angiotensin system, which regulates blood pressure and fluid balance. Elevated ACE levels have been linked to endothelial dysfunction, inflammation, and vascular remodelling, all of which are implicated in the pathogenesis of CAD.^43,44^ Several studies have reported a positive correlation between ACE levels and CAD incidence or severity.^45^ Additionally, ACE inhibitors, which block the action of ACE, have shown beneficial effects in CAD management, further highlighting the potential utility of ACE as a CAD biomarker.^46^ Haptoglobin is an acute-phase protein that binds free haemoglobin to prevent oxidative damage and facilitate its clearance. Dysregulation of haptoglobin levels has been implicated in various inflammatory and oxidative stress-related conditions, including CAD.^47^ Studies have suggested that low levels of haptoglobin are associated with increased CAD risk, possibly due to impaired antioxidant defence mechanisms and enhanced oxidative stress in the vascular system.^48^

The favourable performance of our model on laboratory and clinical data demonstrates its potential to improve CAD risk classification and inform patient management strategies in clinical settings. Additionally, the non-invasive nature of the proposed method, which only requires recording age, sex, and smoking status, and drawing a blood sample, allows the effective and straightforward selection of patients for further diagnostic work-up. These findings demonstrate the model's potential to be applied in clinical practice, offering a useful instrument for assessing patients with suspected CAD and reducing the risks related with invasive procedures. Deploying our CAD prediction model in a clinical environment is a simple endeavour. Healthcare professionals can effortlessly integrate the model into their workflow by inputting the outcomes of standard laboratory examinations. The model’s user-friendly architecture eliminates the need for specialized software, assuring availability for regular implementation in healthcare settings.

This study had several limitations. First, our study utilized visual assessment to define CAD as at least 20% luminal narrowing in major coronary arteries. While it demonstrated promising results in predicting CAD, the absence of quantitative methods like quantitative coronary angiography may affect diagnostic precision. At this initial stage, ML models should be viewed solely as predictive tools rather than diagnostic ones. As more datasets become available for training, we hope to eventually integrate ML algorithms as diagnostic steps in CAD management. The predictive ability of ML algorithms will improve with the increasing volume of patient data. Additionally, our study's reliance on biomarkers such as ACE, haptoglobin, and apolipoprotein A-I, while enhancing predictive accuracy, may limit the model's practical utility due to their inconsistent availability in clinical settings. This constraint could hinder external validation and widespread adoption. Future research should focus on identifying alternative biomarkers with broader accessibility while maintaining predictive performance.

Summing up, our study stands out as the first to develop a computational pipeline utilizing high-quality semi-artificial patient data generated by ML-based approaches to improve CAD detection. Additionally, the extension analysis in YFS affirmed applicability to atherosclerotic vascular disease in a younger population, enhancing the potential of our approach. Our study benefits from a selection of a well-defined, homogeneous patient population from the LURIC cohort. By removing patients with other chronic or acute diseases, confounding variables are reduced, allowing for a more centred analysis of biomarker–CAD relationships. This method yields strong insights into the predictive ability of routine markers. Moreover, the use of mostly routine predictors provides an opportunity for widespread and cost-effective screening for CAD risk, potentially allowing for early identification and intervention in high-risk individuals. Overall, our study contributes to the growing body of literature on CAD risk prediction and may have significant implications for clinical practice.

## Supplementary Material

ztae049_Supplementary_Data

## Data Availability

Data cannot be shared for ethical/privacy reasons.

## Code Availability

To access the code used in this study, please contact the authors directly.
