# Supplementary material for: Predicting early-stage coronary artery disease using machine learning and routine clinical biomarkers improved by augmented virtual data
Source: Eur Heart J Digit Health. 2024 Aug 9;5(5):542–50. doi: 10.1093/ehjdh/ztae049 (PMC11417487; doi:10.1093/ehjdh/ztae049)
Supplement: ztae049_Supplementary_Data [file ztae049_supplementary_data.zip › Supplementary Material.pdf]

Supplementary Materials

Table S1: Inclusion and exclusion criteria

**LURIC inclusion criteria**	**LURIC exclusion criteria**
Caucasian individual of German ancestry living in south-west Germany	any acute illness other than ACS (other acute cardiac diseases, such as decompen-sated heart failure or decompensated valvular disease, or acute non-cardiac diseases, such as infection, endocrine disease or any type of surgery within the previous three months)
except for ACS, individual is in a stable clinical condition (i.e., no concomitant acute illness such as infection or recent accident/surgery)	chronic polymorbid disease where non-cardiac disease predominates (i.e., chronic renal failure and haemodialysis, severe rheumatic arthritis, persistent incapacitation after accident/trauma)
availability of a coronary angiogram (Patient is scheduled for coronary angiography at the time of recruitment into LURIC or a [recent] previous angiogram is available – this inclusion criterium was waived for the individual's family members [mostly siblings] which could be enrolled into LURIC if all other inclusion and exclusion criteria were met.)	history of malignant disease within the previous five years
written informed consent	individuals incapable of understanding the purpose of the study

Supplementary Methods

Evaluation Metrics

These metrics are essential for assessing a binary classification model's performance and offer information about the extent to which it can predict both positive and negative outcomes.

1. Accuracy:
    - The proportion of accurately anticipated cases—both true positives and true negatives—to all instances is known as accuracy. It offers a general evaluation of the model's accuracy.
2. Sensitivity (True Positive Rate):
    - Sensitivity measures how many accurate positive forecasts there are compared to all real positives. It measures how well the model can accurately recognize every real positive case.
3. Specificity:

- The ratio of real negatives to true negative predictions is known as specificity. It shows that the model can correctly detect every real negative case.

4. AUC ROC (Area Under the Receiver Operating Characteristic curve):
   - Based on the receiver operating characteristic curve, AUC ROC assesses the model's ability to distinguish between the positive and negative classes. A higher AUC suggests a more accurate capacity to categorize cases.
5. Precision:
   - Precision is the ratio of true positives to total expected positives. It denotes the model's accuracy in predicting positive cases while minimizing false positives.

Methods to generate virtual data

In the current analysis, we employed two primary approaches for generating virtual patient data. Firstly, we utilized copula functions, which have emerged as a promising alternative for generating appropriate combined virtual variables. These methods involve constructing a multivariate cumulative distribution function (CDF) in which the marginal probability distribution of variables is uniform.[1] Copulas enables the separation of the interdependence of the multivariate random vector from its marginal distribution.

Let X ∈ $\mathbb{R}^P$, the random vector considered. Its joint distribution function can be written as:

$$H\left(x_1, x_2, \ldots., x_p\right) = C\left(F_1(x_1), F_2(x_2), \ldots., F_p\left(x_p\right)\right) X^T \in \mathbb{R}^P \ , \quad (1)$$

where $C: [0,1]_p \rightarrow [0,1]$ is the copula and F_i denotes the ith marginal distribution function.

By turning Eq. (1) around, we can project any MVD to the unit square to recover the copula *C.*

$$C\left(u_1, u_2, \ldots., u_p\right) = H\left(F_1^{-1}(u_1), F_2^{-1}(u_2), \ldots.., F_p^{-1}\left(u_p\right)\right), \quad (2)$$

where F_i^{-1} is the inverse marginal distribution function of F_i.

There are two categories of copulas: Archimedean and elliptical copulas. Due to their simplicity and properties, Archimedean copula families are effective for modeling bivariate distributions.[2] However, in this study, we employed elliptical copulas as they can generate and simulate random variables with high dimension. The Gaussian Copula with a density, described by Eq. (3), is produced by projecting the multivariate distributions.[3]

$$C_r(u) = \frac{1}{\sqrt{|r|}} exp(-\frac{1}{2} F^{-1^T} (r^{-1} - I) F_i^{-1}) , \quad (3)$$

where $r \in [-1, 1]_{\mathrm{pxp}}$ is the correlation matrix with 1 in the diagonal and I is the identity matrix. [4]

As a second approach we used a GAN-based Deep Learning data synthesizer (CTGAN). CTGAN a conditional generator to address challenges such as: (i) data that is mixed (discrete and continuous columns), (ii) discrete columns may occasionally be imbalanced and cause modeling difficulties, while continuous columns may have several modes. CTGAN, a synthetic tabular data generator, was used in this study. Additionally, we utilized its variant adapted with variational autoencoder (VAE) for generating mixed-type tabular data, known as TVAE.[5] Both techniques were applied in the present research.

Supplementary Results

Table S2: Performance Metrics for Each Fold, Mean Values, and 95% Confidence Intervals: Random Forests (RFs)

Fold	Accuracy	Sensitivity	Specificity	AUC ROC	Precision
**1**	0.78	0.88	0.72	0.85	0.78
**2**	0.79	0.90	0.71	0.86	0.80
**3**	0.80	0.89	0.69	0.87	0.79
**4**	0.77	0.87	0.70	0.84	0.78
**5**	0.81	0.88	0.71	0.88	0.80
**6**	0.78	0.89	0.68	0.85	0.78
**7**	0.80	0.90	0.69	0.86	0.80
**8**	0.79	0.88	0.72	0.87	0.79
**9**	0.78	0.89	0.70	0.85	0.79
**10**	0.80	0.88	0.69	0.87	0.79
**Mean**	0.79	0.89	0.70	0.86	0.79
**CI (95%)**	0.79 ± 0.01	0.89 ± 0.01	0.70 ± 0.02	0.86 ± 0.01	0.79 ± 0.01

Table S3: Performance Metrics for Each Fold, Mean Values, and 95% Confidence Intervals: Gradient Boosting (GB) Algorithms

Fold	Accuracy	Sensitivity	Specificity	AUC ROC	Precision
**1**	0.79	0.88	0.75	0.86	0.81
**2**	0.80	0.86	0.75	0.87	0.82
**3**	0.81	0.88	0.74	0.88	0.83
**4**	0.78	0.87	0.72	0.85	0.80
**5**	0.82	0.86	0.75	0.89	0.83
**6**	0.79	0.87	0.74	0.86	0.81
**7**	0.80	0.88	0.73	0.87	0.82
**8**	0.81	0.86	0.74	0.88	0.83
**9**	0.79	0.87	0.75	0.86	0.81
**10**	0.80	0.86	0.74	0.87	0.82
**Mean**	0.80	0.87	0.74	0.87	0.82
**CI (95%)**	0.80 ± 0.01	0.87 ± 0.01	0.74 ± 0.01	0.87 ± 0.01	0.82 ± 0.01

Supplementary Figure legends

Figure S1

Spearman Rank-Order Correlation Matrix. Each cell in the matrix indicates the correlation coefficient between two attributes, which ranges from -1 (perfect negative correlation) to 1 (perfect positive correlation). A value near to zero suggests a lower association. The visualization shows the strength of the correlations between the real (left) and virtual datasets (right), making it easier to examine their relationships.

Figure S2

Comparative analysis of feature distributions, highlighting original (real) and synthetic data using density histograms.

Figure S3

Comparative analysis of feature distributions using kernel density estimation, showcasing the differences between original (real) and synthetic data.

Figure S4

Comparative analysis of feature distributions using violin plots, highlighting differences between original (real) and synthetic data.

References

1 Sklar A. Random variables, joint distribution functions, and copulas. Kybernetika 1973; 09: (449)-460.

2 Li H, Wang D, Singh VP, Wang Y, Wu J, Wu J et al. Non-stationary frequency analysis of annual extreme rainfall volume and intensity using Archimedean copulas: A case study in eastern China. J Hydrol 2019; 571: 114–131.

3 Arbenz P. Bayesian Copulae Distributions, with Application to Operational Risk Management—Some Comments. Methodol Comput Appl Probab 2013; 15: 105–108.

4 An Introduction to Copulas. Springer: New York, NY, 2006.

5 Xu L, Skoularidou M, Cuesta-Infante A, Veeramachaneni K. Modeling Tabular data using Conditional GAN. 2019.
