# Supplementary figures and images for: Predicting early-stage coronary artery disease using machine learning and routine clinical biomarkers improved by augmented virtual data
Source: Eur Heart J Digit Health. 2024 Aug 9;5(5):542–50. doi: 10.1093/ehjdh/ztae049 (PMC11417487; doi:10.1093/ehjdh/ztae049)

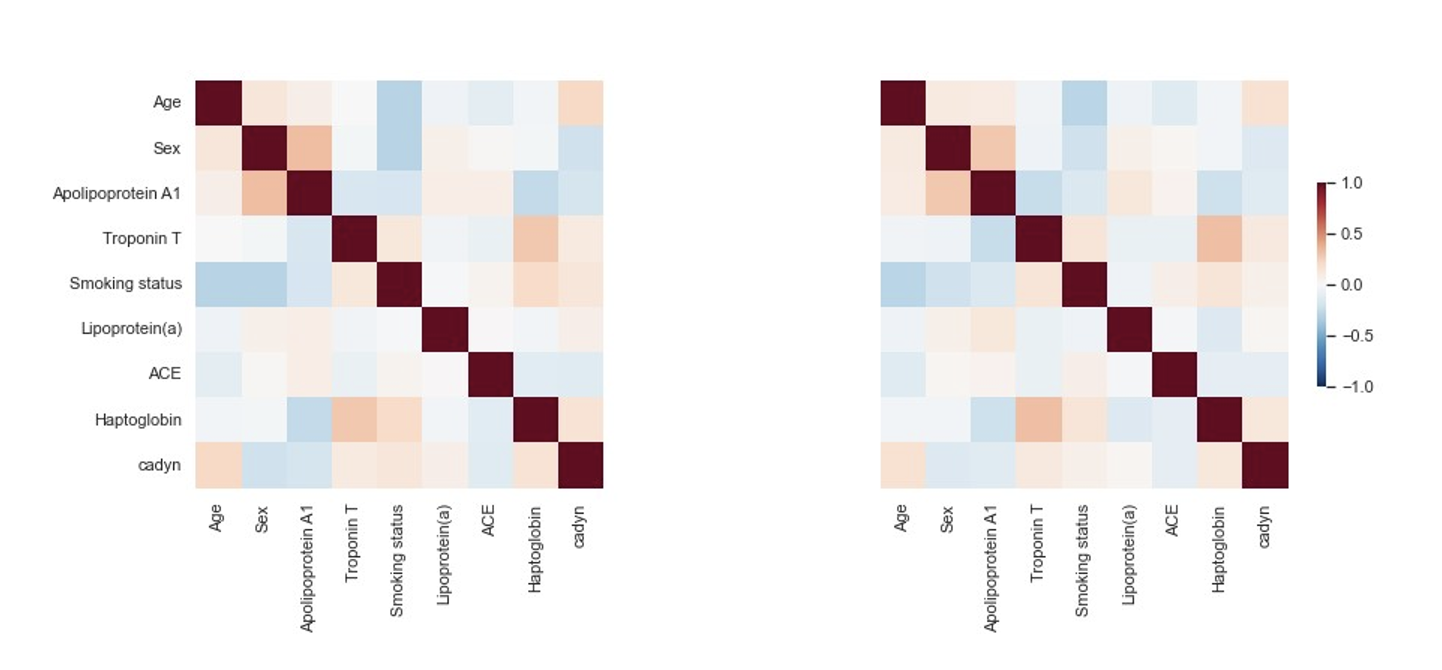

Supplement: ztae049_Supplementary_Data [file ztae049_supplementary_data.zip › Supplementary Figure S1.tiff]

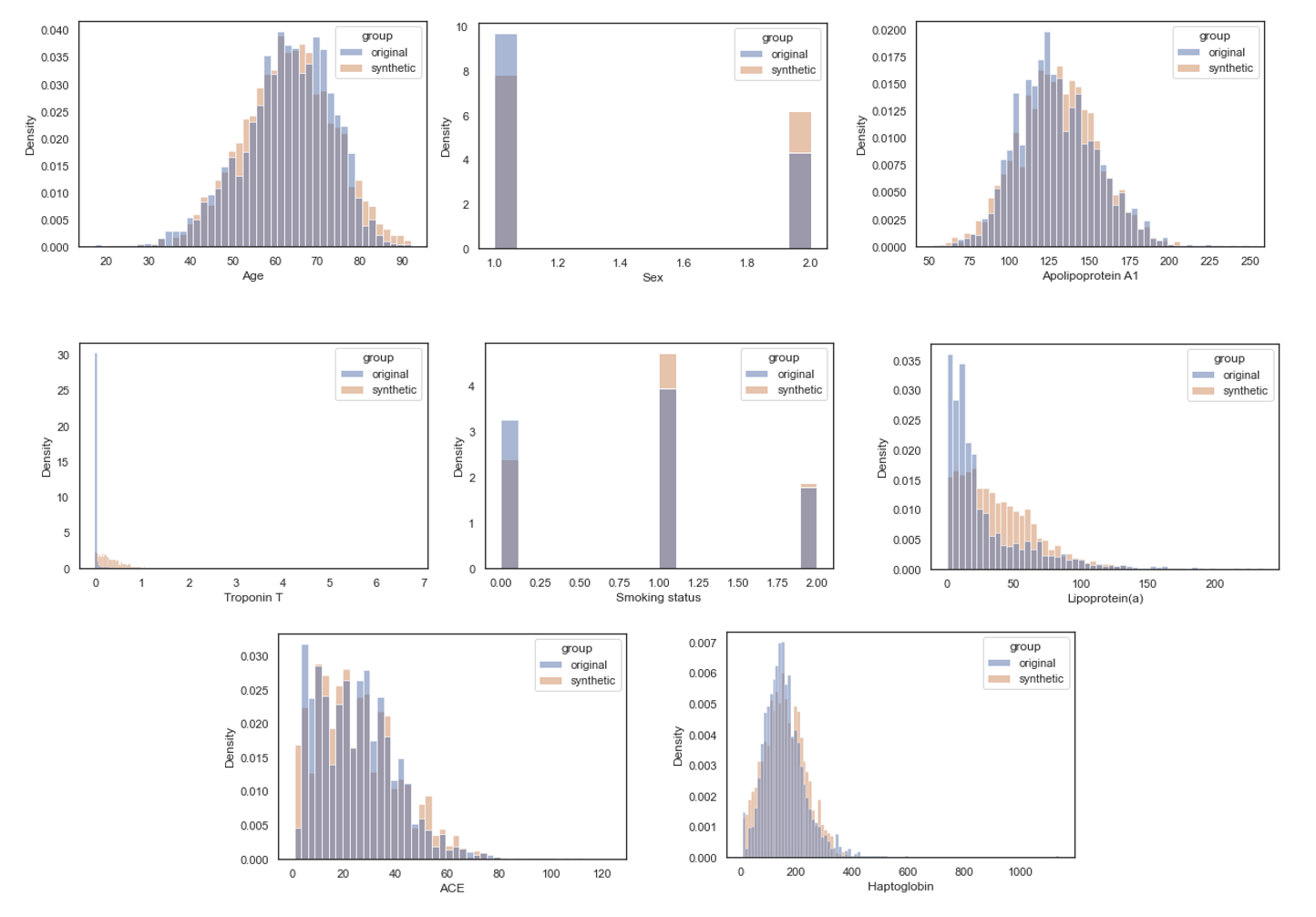

Supplement: ztae049_Supplementary_Data [file ztae049_supplementary_data.zip › Supplementary Figure S2.tiff]

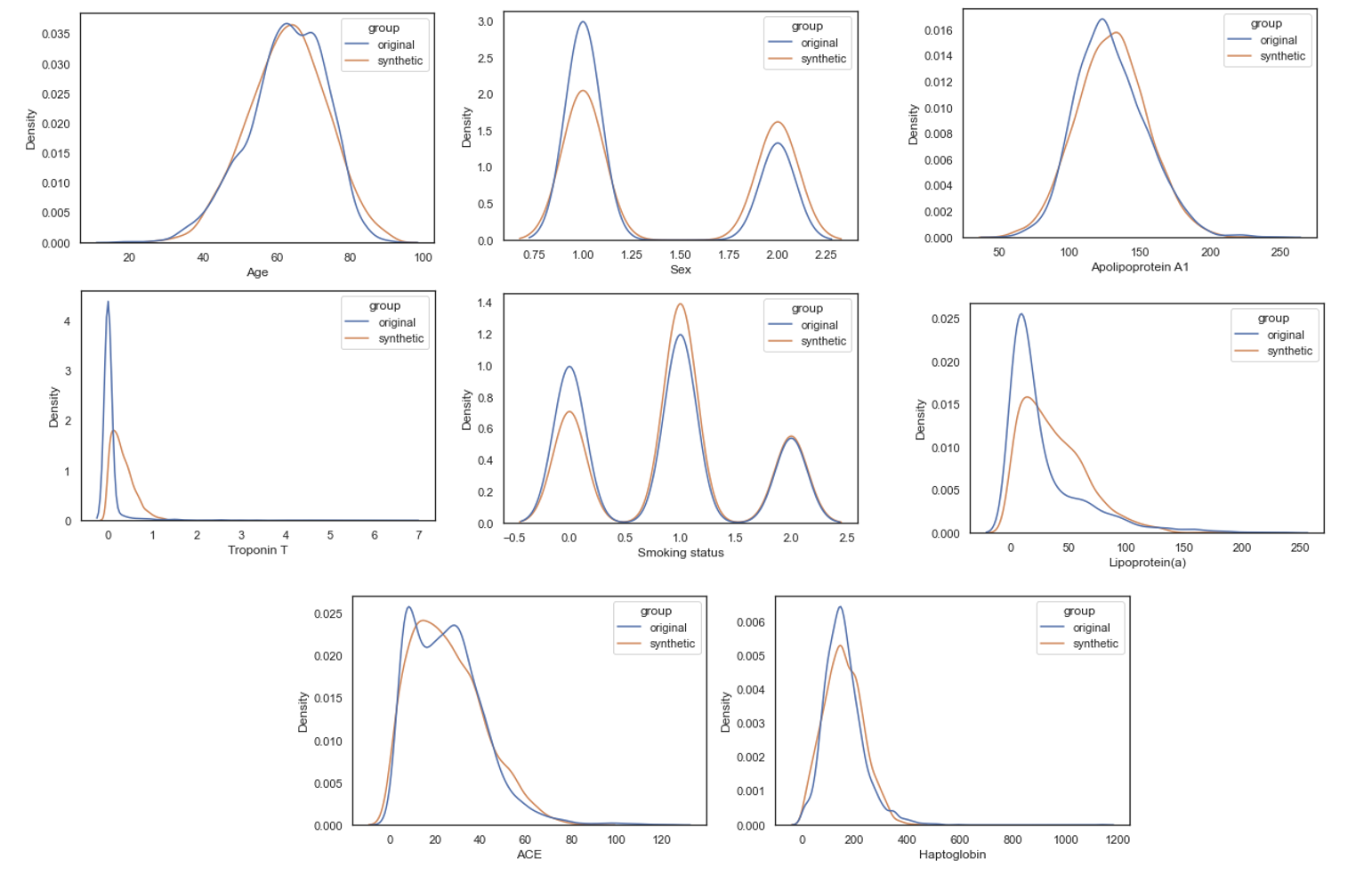

Supplement: ztae049_Supplementary_Data [file ztae049_supplementary_data.zip › Supplementary Figure S3.tiff]

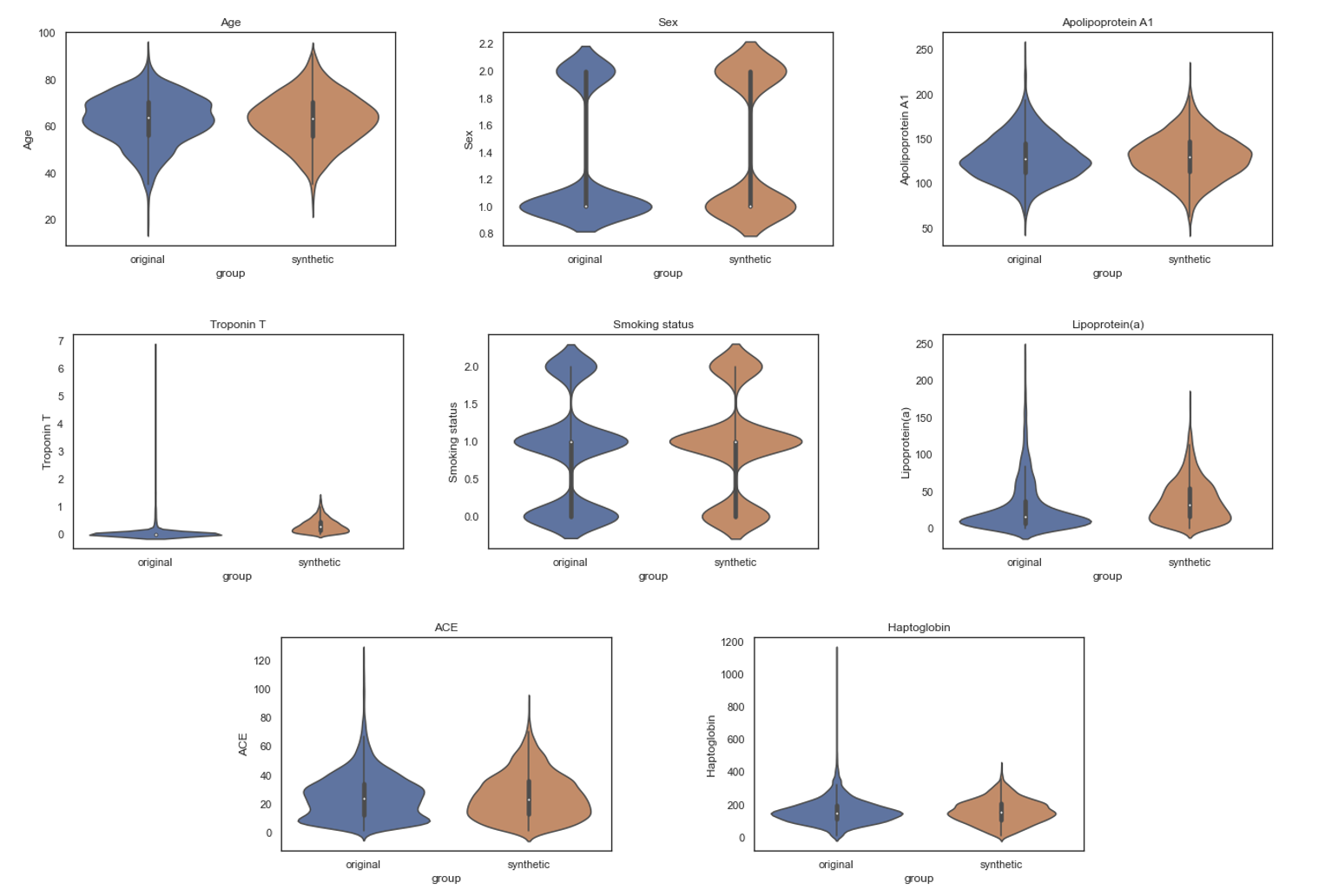

Supplement: ztae049_Supplementary_Data [file ztae049_supplementary_data.zip › Supplementary Figure S4.tiff]
